# A Distinct Layer of the Medulla Integrates Sky Compass Signals in the Brain of an Insect

**DOI:** 10.1371/journal.pone.0027855

**Published:** 2011-11-16

**Authors:** Basil el Jundi, Keram Pfeiffer, Uwe Homberg

**Affiliations:** 1 Department of Biology, Animal Physiology, Philipps-University, Marburg, Germany; 2 Department of Physiology and Biophysics, Dalhousie University, Halifax, Canada; University of Arizona, United States of America

## Abstract

Mass migration of desert locusts is a common phenomenon in North Africa and the Middle East but how these insects navigate is still poorly understood. Laboratory studies suggest that locusts are able to exploit the sky polarization pattern as a navigational cue. Like other insects locusts detect polarized light through a specialized dorsal rim area (DRA) of the eye. Polarization signals are transmitted through the optic lobe to the anterior optic tubercle (AOTu) and, finally, to the central complex in the brain. Whereas neurons of the AOTu integrate sky polarization and chromatic cues in a daytime dependent manner, the central complex holds a topographic representation of azimuthal directions suggesting a role as an internal sky compass. To understand further the integration of sky compass cues we studied polarization-sensitive (POL) neurons in the medulla that may be intercalated between DRA photoreceptors and AOTu neurons. Five types of POL-neuron were characterized and four of these in multiple recordings. All neurons had wide arborizations in medulla layer 4 and most, additionally, in the dorsal rim area of the medulla and in the accessory medulla, the presumed circadian clock. The neurons showed type-specific orientational tuning to zenithal polarized light and azimuth tuning to unpolarized green and UV light spots. In contrast to neurons of the AOTu, we found no evidence for color opponency and daytime dependent adjustment of sky compass signals. Therefore, medulla layer 4 is a distinct stage in the integration of sky compass signals that precedes the time-compensated integration of celestial cues in the AOTu.

## Introduction

**Figure 1:**
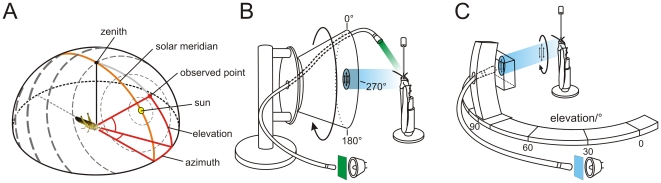
Sky polarization pattern and experimental setups. (A) Polarization pattern of the blue sky at a solar elevation of 40°. Grey bars show *E*-vector orientation and degree of polarization (thickness of bars). The zenithal direction is shown as a black line, the solar meridian is illustrated in orange. The red spot indicates an observed point in the sky at an elevation of 45° and an azimuth of 20°. (B) Stimulus device used to analyze polarization sensitivity and azimuthal response to monochromatic unpolarized stimuli. The animal was stimulated with zenithal polarized light produced by a blue LED (470 nm) that passed a rotating polarization filter. In addition, an unpolarized green (530 nm) or UV (350 nm) light spot provided by light from a xenon lamp was moved in clockwise and counter clockwise directions around the head at an elevation of about 45°. The stimuli were presented successively during recordings from the neurons. (B) Experimental device used for measuring the bilateral extent of the receptive field across the left-right meridian. Monochromatic blue light (450 nm) provided through a light guide and a rotating polarizer was delivered from a perimeter that allowed the presentation of polarized light from different elevations along the left-right meridian.

Many insects show impressive navigational skills during homing and seasonal migrations [1], [2]. The sun is often the most important directional cue. It is the brightest spot in the sky and gives rise to a characteristic pattern of polarization (Fig. 1A) and chromatic contrast across the sky. All of these features may serve as references to determine azimuthal directions [3].

Desert locusts *(Schistocerca gregaria)* migrate in huge swarms throughout North Africa and the Middle East. Behavioral experiments on tethered flying locusts suggest that they are able to perceive the plane of sky polarization [4]. Like other insects, desert locusts detect polarized light with photoreceptors located in a specialized dorsal rim area (DRA) of the compound eye [5], [6]. DRA photoreceptors are specifically adapted for high polarization sensitivity and transmit polarization information to dorsal rim areas of the lamina and medulla in the optic lobe. In the brain, polarization-sensitive (POL) interneurons respond with sinusoidal modulation of firing rate during zenithal stimulation with light passing through a rotating polarizer [7], [8]. Most POL-neurons are maximally excited at a particular *E-*vector orientation (Φ_max_) and are maximally inhibited at an orthogonal orientation, termed Φ_min_ (polarization opponency).

In the locust brain, polarized light signals are processed in distinct neuropils [9]. Small field transmedulla neurons, previously termed “line tangential neurons” [9], [10], ramify in the dorsal rim area of the medulla (DRMe) and transfer polarization signals via the lobula to the anterior optic tubercle (AOTu). Second-order interneurons continue from the AOTu to the lateral accessory lobe [10], [11]. Signals are, finally, processed in the central complex [12], which holds a topographic representation of zenithal *E*-vectors and may, thus, act as an internal sky compass [13]. Output neurons from the central complex project again to the lateral accessory lobe [12], [14] and, finally, polarization information is sent via descending neurons to thoracic motor control centers [15].

In addition to polarization signals, POL-neurons of the AOTu respond to other celestial cues such as the color and azimuth of a light spot, which might allow them to distinguish between the solar and antisolar sky hemispheres [8], [16]. Moreover, these neurons show a daytime-dependent adjustment in *E*-vector tuning suited to compensate for daytime changes in solar elevation.

Whereas the neural mechanisms underlying sky compass navigation in the locust central brain have been studied in detail [8], [11]–[13], [16], neural circuits in the optic lobe that mediate integration of different orientation signals are virtually unexplored. In particular, the origin of polarization opponency, the site of convergence of celestial compass signals, and the site and mechanism of daytime compensation for changes in solar elevation are unknown. To address these issues, we analyzed POL-neurons in the medulla of the locust optic lobe. All POL-neurons ramified in the same medulla layer, and most of them had sidebranches in the accessory medulla. Beside polarized light, neurons responded to unpolarized green or UV light spots that moved around the locust head. In contrast to neurons of the AOTu, no evidence was found for a daytime-dependent compensation of solar elevation in the responses of the neurons, suggesting that time compensation occurs at a stage between the medulla and the AOTu of the brain.

## Materials and Methods

### Animals and preparation

Experiments were performed on sexually mature desert locusts (*Schistocerca gregaria)*. Animals from our laboratory colony were reared under crowded conditions at 28°C in a 12 h light/dark cycle. Some locusts from this colony were raised in a greenhouse and had direct view of the sky. Experiments on the greenhouse reared animals were performed between August 26 and September 30. Animals were cold anesthetized (at least 30 min), their legs and wings were removed, and their mouthparts were closed with wax. The locusts were fixed anterior uppermost to a metal holder, and a ridge of wax was attached frontally from the mouthparts to the anterior edge of the compound eyes. After opening the head capsule, fat, trachea and muscles above the brain were removed. To reduce hemolymph pumping, the abdomen was opened posteriorly, the intestine was removed, and the abdomen was constricted with a tightly knotted thread. The brain was stabilized further by a wire platform inserted between the esophageal connectives. To facilitate electrode penetration, the neural sheath above the optic lobe was removed. During animal preparation and recording of neural activity the brain was immersed in locust saline [17]. A silver wire inserted into the hemolymph/saline in the opened head capsule was used as the reference electrode.

### Electrophysiology

For intracellular recordings, sharp glass electrodes (resistance: 60–200 MΩ) were drawn from borosilicate capillaries (inner diameter: 0.75 mm; outer diameter: 1.5 mm; Hilgenberg, Malsfeld, Germany) with a Flaming/Brown horizontal puller (P-97, Sutter, Novata, CA, USA). The tips of the electrodes were filled with 4% Neurobiotin (Vector Laboratories, Burlingame, UK) in 1 M KCl and their shanks, with 1 M KCl. Neural signals were amplified (10×) with a custom-built amplifier and monitored by a custom-built audiomonitor and a digital oscilloscope (Hameg HM 205–3, Frankfurt/Main, Germany). A CED 1401 plus interface (Cambridge Electronic Design, UK) was used to sample the signals at a rate of 5 kHz. The sampled signals were stored on a computer using the software Spike2 (version 6.02; Cambridge Electronic Design). To identify the recorded neuron Neurobiotin was injected iontophoretically with constant depolarizing current (2–3 nA).

### Visual stimulation

Two visual stimulus devices were used (Fig. 1B,C). The first device allowed us to test the neural responses to polarized blue light and to unpolarized chromatic light spots (Fig. 1B). Linearly polarized light from the zenith was produced by passing the light of a blue LED (Luxeon LED emitter, LXHL-BB01, 1 W, 470 nm, Philips Lumileds Lighting Company, San Jose, CA, USA) through a rotating polarizer (HNP'B, Polaroid, Cambridge, MA, USA). The angular extent of the stimulus at the locust eye was ∼5.4°. The polarizer was rotated in clockwise (0°–360°) and counter clockwise (360°–0°) directions with an angular velocity of 30°/s. In addition, an unpolarized green (530 nm) or UV (350 nm) light spot produced by a xenon arc (XBO 150W, LOT-Oriel Group, Darmstadt, Germany) and passed through a light guide, interference filters, and a circular neutral density wedge moved at an elevation of about 45° around the center of the locust's head. Unpolarized light was presented through a quartz light guide (Schott Fiber Optics, Mainz, Germany). Photon flux for both stimuli was adjusted to 2.3×10^13^ photons/cm^2^s. The angular size of the unpolarized light spot was ∼3.8°, movement velocity around the head was 30°/s.

With the second stimulation device polarized blue light was presented at different elevations of the visual field to analyze the receptive field structure across the left-right meridian (Fig. 1C). Polarized blue light (450 nm, photon flux 8.1×10^12^ photons/cm^2^s) was obtained from a xenon arc and passed through a light guide (Schölly Fiberoptic, Denzingen, Germany) and a linear polarizer (HNP'B, Polaroid, Waltham, MA, USA). The light guide and the polarization filter were attached to a perimeter that allowed us to present polarized light through the rotating polarizer from different elevations (rotational speed 30°/s, 360° clockwise and counter clockwise rotations). The angular size of the stimulus at the locust eye was ∼4.7°. Ocular dominance was tested by shielding one eye from the light source with a handheld piece of cardboard during dorsal polarized light stimulation. The terms ipsilateral and contralateral stimulation are defined with respect to the position of the soma of the recorded neuron in the brain. An *E*-vector orientation of 0° was defined as being parallel to the body axis of the animal.

### Immunocytochemistry

After injection of Neurobiotin into the recorded neurons, brains were dissected from the head and were fixed in a solution of 4% paraformaldehyde, 0.25% glutaraldehyde, and 2% saturated picric acid in 0.1 M phosphate buffer (Neurobiotin fixative) overnight at 4°C. Afterwards, brains were rinsed 1 h with 0.1 M phosphate buffered saline (PBS, pH 7.4) and were then incubated for at least 3 days with streptavidin conjugated to Cy3 (1∶1000; Cy3-streptavidin; Dianova, Hamburg, Germany) in 0.1 M phosphate buffered saline containing 0.3% Triton X-100 (PBT). The brains were then rinsed two times in 0.1 M PBT, followed by rinses in 0.1 M PBS and were dehydrated in an ascending ethanol series (25%–100%, 15 min each). After treatment in an ethanol/methyl salicylate (1∶1, 15 min) solution, brains were cleared in methyl salicylate for 35 min and, finally, mounted in Permount (Fisher Scientific, Pittsburgh, PA, USA) between two glass coverslips. To prevent compression of the brains, ten reinforcement rings (Zweckform, Oberlaindern, Germany) were used as spacers.

For detailed anatomical analysis of selected neurons, brains were rehydrated and sectioned as described in detail by [18]. Briefly, the embedding medium was removed by incubation in xylene (2–4 h). Brains were rehydrated in a descending ethanol series and were embedded in gelatine/albumin overnight at 4°C. The brains were then sectioned in frontal plane at 130 or 250 µm with a vibrating-blade microtome (Leica VT1200 S, Leica Microsystems, Wetzlar, Germany). They were preincubated with 5% normal goat serum (NGS; Jackson ImmunoResearch ,Westgrove, PA, USA) in 0.1 M PBT overnight at 4°C and were then incubated for 6 days with a monoclonal mouse antibody against synapsin I (SYNORF1, dilution 1∶50; [19]; kindly provided by Dr. E. Buchner, Würzburg) and with Cy3-streptavidin (1∶1000) in 0.1 M PBT containing 1% NGS. After incubation with the secondary antibody, goat anti-mouse conjugated to Cy5 (Cy5-GAM, 1∶300; Jackson ImmunoResearch, Westgrove, PA, USA) and with Cy3-streptavidin (1∶1000) in 1% NGS and 0.1 M PBT for 4 d at 4°C, sections were dehydrated, cleared and embedded in Permount between two coverslips.

### Tracer application

For tracing of neuronal pathways, a piece of cuticle was removed from the frontal part of the head capsule. Air sacs and trachea were removed to expose the brain. A small piece of the neural sheath above the target area was removed with forceps. Glass micropipettes were drawn from borosilicate glass and broken to a tip diameter of 10–50 µm. The tip of the pipette was dipped into petroleum jelly and then into biotinylated dextran (3000 MW, lysine fixable, Invitrogen, Eugene, OR, USA) under visual control to confirm that a few tracer crystals were attached to the petroleum jelly. The pipette was attached to a block of plasticine and was manually inserted into the target area. Residual, superficial tracer was removed by extensive rinses with locust saline. The head capsule was then closed by replacing the previously removed piece of cuticle.

For tracing of photoreceptor axons from the dorsal rim area, the cornea and crystalline cones of dorsal rim ommatidia were removed and a drop of dextran conjugated to Alexa fluor 488 (10,000 MW, anionic; fixable; Invitrogen) was applied. The eye was sealed with petroleum jelly. Animals were kept overnight at 4°C in a moist chamber to allow tracer uptake and distribution. The next day, brains were dissected out of the head capsule and were fixed in Neurobiotin fixative at 4°C overnight. Subsequently, brains were rinsed for 1 h with 0.1 M PBS at room temperature and were treated with 1 mg/ml collagenase-dispase (in 0.05 Tris-HCl, pH 7.6) for 1 h. After additional rinsing with 0.1 M PBT for 2 h (6×20 min) the brains were preincubated (4°C, overnight) with 5% NGS in 0.1 M PBT containing 0.02 % sodium azide. Subsequently, the wholemount preparations were incubated (4–6 days at 4°C) with anti-synapsin I (1∶50) and with Cy3-streptavidin (1∶1000) in 0.1 M PBT containing 1% NGS and 0.02 % sodium azide. After extensive rinsing, the brains were incubated with Cy5-GAM (1∶300) and Cy3-streptavidin (1∶1000) in 0.1 M PBT, 1% NGS and 0.02 % sodium azide for up to three days at 4°C. After rinsing, preparations were dehydrated, cleared and mounted in Permount.

### Image acquisition, processing and 3D reconstruction

Brain sections were scanned with a confocal laser scanning microscope (CLSM, Leica TCS SP5) using a 20×(HCX PL APO 20×/0.70 Imm UV, working distance: 260 µm; Leica) or a 40×(HCX PL APO 40×/1.25 Oil UV, working distance: 100 µm; Leica) oil objective. The Cy3 signal was scanned by using a DPSS (561 nm) laser and Cy5-fluorescence was detected with a HeNe (633 nm) laser. All neurons were scanned in several image stacks with a resolution of 1024×1024 (voxel size: 0.1–0.5×0.1–0.5×0.5–1.5 µm).

The AOTu-injected wholemount preparations were scanned at 1024×1024 pixel resolution with a 10×(HC PL APO 10×/0.40 Imm CS, working distance: 360, Leica) oil objective and with a voxel size of 1×1×3 µm. In addition to Cy3- and Cy5-fluorescence, the Alexa-488 signal was detected with an Ar (488 nm) laser. As a result of the thickness of the wholemount preparations the brains were scanned from anterior and posterior.

The obtained image stacks were processed on a personal computer using Amira 4.1.2 or 5.2.1 software (Visage Imaging, Fürth, Germany). The procedure for aligning corresponding image stacks and the three dimensional (3D) reconstructions of brain areas based on anti-synapsin staining were described in detail in [20]. 3D reconstructions of the neurons were performed using the Amira add-on tool *SkeletonTree* generated by [21]. For the reconstruction of a neuron, corresponding image stacks were not merged but were oriented with respect to each other. The neuron was reconstructed by opening the image stacks consecutively and labeling the particular part of the neuron. Volume rendering visualization of the AOTu injected brains were displayed in Amira 4.1.2. To reduce background staining and to visualize only the region of interest in the central brain, the image stack was masked using the module ‘Arithmetic’.

### Data analysis

Spike trains were evaluated by a script in Spike2, written by one of the authors (KP). To study the neural response to polarized and unpolarized light, events during the 360° rotations were detected through threshold-based event detection and were assigned to the particular *E*-vector orientation during rotation of the polarizer or to the corresponding angle during circling of the unpolarized light spot. These angles were then analyzed statistically for significant difference from randomness using Oriana 2.02 software (Kovach Computing Services, Anglesey, UK). Responses of neurons to polarized light were analyzed through the Rayleigh test for axial data whereas the neural activities during stimulation with the moving unpolarized light spots were examined using the Rayleigh test for circular data. If the distribution of angles was significantly different from randomness (α = 0.05) the corresponding mean angle of the distribution was defined as the preferred *E*-vector orientation (Φ_max_) or as the preferred azimuthal direction of the neuron. The distribution of the preferred orientations of different recordings from the same neuron type was analyzed statistically using Rao's spacing test [22]. The clustering of the distribution of the preferred orientations around the averaged Φ_max_ of the neurons was analyzed through the length of the mean vector *r* (Oriana). The *r* value ranges from 0 to 1; a value closer to 1 indicates that the observations are clustered more closely around the mean Φ_max_ than with a lower value. To determine whether the mean angles of two samples differed significantly from each other, we used the Watson-Williams F-test for circular data. Mean background activities of the neurons were measured by spike counts during time periods of the recordings without stimulation and current injection. The mean frequency during stimulation was visualized using a moving average algorithm (bin size: 1s).

To quantify and compare the neural response strength at different elevations across the left-right meridian or during stimulation of only one eye, the response amplitude value R was calculated [23]. To that end the stimulation period was divided into 18 bins (each 20° wide), and for each bin the difference between the specific spike frequency and the mean frequency during the whole stimulation period was calculated. The sum from all bins was defined as the response value R. The widths of the receptive fields were defined by the half-maximal response amplitude compared to the background activity. Circular plots of the mean activity of the neurons, receptive field plots, and ocular dominance diagrams were created in Origin 6.0 (Microcal, Northhampton, CA).

### Model calculations

Receptive fields were modeled by creating a circular raster of sample points with a diameter of 110° for MeMe1 neurons and 90° for TML1 neurons. This corresponds to the lateral extent of their visual fields with at least 50% response strength. Calculation of the raster was carried out in two steps, similar to the method described by [24]. In the first step, we created a zenith-centered raster. Sample points were distributed on circles of equal latitude with the difference in latitude between two circles being 2°. The circumference *c* of each circle was given by
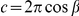
where *β* = latitude of the respective circle. The number of points on each circle *n* was calculated as
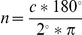



The distance *d* between two points on each circle of latitude was



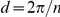
.

In the second step, this zenith-centered raster was moved to the appropriate location in space by vector transformation.

For each sample point we then calculated the *E*-vector and the degree of polarization according to the single scattering Rayleigh model [25] as described by [16] and [26]. While the natural polarization pattern of the sky follows the Rayleigh model rather well in terms of *E*-vector orientation [27], the degree of polarization *(d)* is usually much lower and even under optimal conditions does not exceed 0.75 [28]. We therefore multiplied the *d* value by 0.75. The longitudinal axis of the animal was defined as being parallel to the 0°–180° meridian. Calculations were done for a solar azimuth set to the preferred direction of green light (Φ_max_green) of the respective neuron. Solar elevation was varied between 0° and 90° in steps of 1°. For each step, the average *E*-vector orientation within the visual field was calculated from the individual *E*-vectors at each raster point, weighted by the respective degree of polarization. This is equivalent to calculating the second order mean angle as described by [22]. The mean degree of polarization was calculated as the arithmetic mean of the individual degrees of polarization at each point within the visual field. Mean *E*-vector and mean degree of polarization were plotted against the elevation of the sun.

## Results

### Polarization-sensitive neurons in the optic lobe

To analyze the processing of polarized light signals in the optic lobe, neurons of the medulla were studied through intracellular recordings combined with dye injections. For detailed anatomical analysis the recorded neurons were imaged and were reconstructed in three dimensions. In addition to transmedulla neurons that send polarization vision information from the optic lobe into the central brain [10], we identified three major classes of polarization-sensitive (POL) neurons in 57 recordings.

### Tangential intrinsic medulla neurons

**Figure 2:**
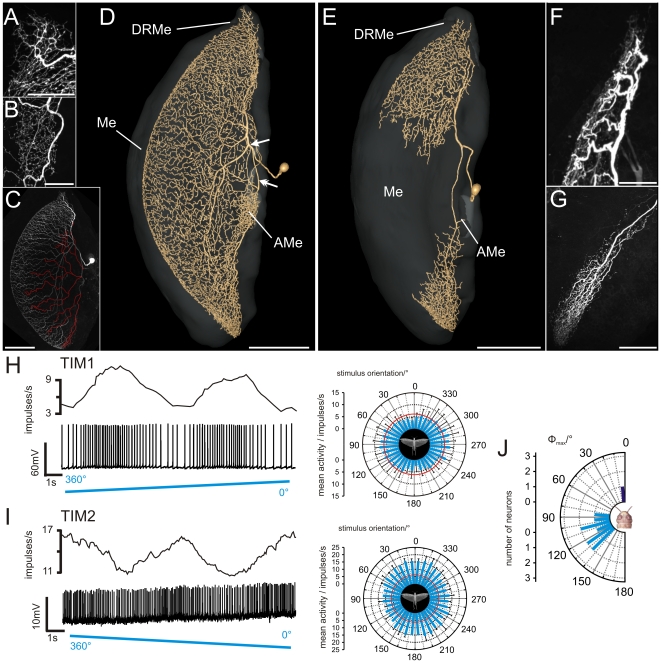
Morphology and physiology of tangential intrinsic medulla neurons (TIM1, TIM2). (A) Ramifications of a TIM1 neuron in the dorsal rim area of the medulla, maximum intensity view of confocal images. (B) Maximum intensity projection of the arborizations of a different TIM1 neuron in the accessory medulla. (C) Maximum intensity visualization of a TIM1 neuron (same neuron as shown in A) illustrates varicose and, thus, potential output regions (labeled in red) that originate from a ventrally projecting neurite. (D) Three-dimensional reconstruction of a TIM1 neuron within the medulla (Me, transparent), anterolateral view. After entering the medulla, the primary neurite splits into two main fibers. One collateral (arrow) projects dorsally, the other one (double arrow) projects to the accessory medulla (AMe) and the ventral medulla. DRMe, dorsal rim area of the medulla; AMe, accessory medulla. (E) Ramifications of a TIM2 neuron in the medulla (Me) reconstructed in three dimensions. Neuropils are shown in transparent grey, anterolateral view. (F) Arborizations of the TIM2 neuron in dorsal regions of the medulla and in the DRMe, maximum intensity projection of confocal image stack. (G) Maximum intensity projection of ramifications of TIM2 in the ventral medulla. The arborizations were more varicose. (H) Firing rate of TIM1 neuron, shown in A and G, during stimulation with polarized blue light. The polarizer was rotated 360° in clockwise direction. Upper trace: mean spike frequency during stimulation (moving average of spike rate in window size 1s); lower trace: spike train; right plot: circular diagram of mean spiking frequency plotted against *E-*vector orientation (bin size: 10°; n = 6; error bars = standard deviation, Φ_max_ = 99°, Rayleigh test, p = 2.45×10^−5^). Red circle indicates background activity of the neuron. (I) Mean spiking frequency (upper trace, moving average of spike rate in 1s time windows) and spike train (lower trace) of the TIM2 neuron shown in D stimulated with polarized light. The polarizer rotated 360° in counter clockwise direction. Right panel: mean spike activity and background activity (red circle) of the TIM2 neuron from ten 360° rotations of the polarization filter (bin width: 10°, error bars = SD, Φ_max_ = 4°, Rayleigh test, p = 0.003). (J) Distribution of the mean preferred orientations of TIM1 neurons (n = 9, bright blue) and the TIM2 neuron (n = 1, dark blue) (bin size: 10°). The Φ_max_ values were calculated from equal numbers of clockwise and counter clockwise rotations of the polarizer. All TIM1 neurons were analyzed in the medulla of the left brain hemisphere. The distribution of Φ_max_ orientations of the recorded TIM1 neurons was significantly different from randomness (mean Φ_max_ angle: 113°±18° (SD), Rao's spacing test, p<0.01; length of mean vector *r* = 0.819). Scale bars: (A,B,E) 50 µm; (G) 100 µm; (C,D,E) 200 µm.

The first class of POL-neurons, termed tangential intrinsic medulla neurons (TIM), had ramifications in the medulla and in the dorsal rim area of the medulla (DRMe, Fig. 2, Movie S1). The tangential intrinsic medulla neuron 1 (TIM1) was studied in 9 experiments and was already introduced by [29]. The morphologies of the 9 neurons were indistinguishable suggesting that all recordings were from the same neuron. It ramified through an entire layer of the medulla and arborized additionally in the DRMe (Fig. 2A–D) and in the accessory medulla (AMe, Fig. 2B) a small neuropil at the anterior-median edge of the medulla that in cockroaches and flies serves as the master circadian clock in the brain [30]–[32]. Its soma was located anteromedially from the medulla, and its primary neurite entered the medulla at its median proximal edge (Fig. 2C,D). After entering the medulla, the primary neurite split into two main neurites. One collateral (arrow in Fig. 2D) projected dorsally, ramified in the DRMe and gave off several large sidebranches that arborized throughout a single layer of the medulla (white processes in Fig. 2C). The second collateral (double arrow in Fig. 2D) projected ventrally within the same medulla layer and arborized, in addition, extensively in the AMe. Sidebranches originating from the ventral collateral extended widely through the entire medulla layer (red arborizations in Fig. 2C), similar to the ramifications from the dorsal collateral. Owing to the complex and uniform branching pattern, identification of possible input and output regions of TIM1 was difficult. Sensitivity to zenithal polarized light (see below) suggested that the neuron received input via its dorsal collateral. The dense arborizations in the AMe were the least varicose parts of the neuron (Fig. 2B) and might be a second input region. Arborizations of the ventral collateral were varicose, and therefore, possibly output regions.

TIM1 neurons responded with polarization opponency to polarized light from dorsal direction, i.e. they were maximally activated at a particular *E-*vector orientation (Φ_max_) and were maximally inhibited at an orthogonal *E*-vector (Φ_min_) (Fig. 2H). The neurons had a mean background spiking activity of 14.2±7.9 (SD) impulses/s and an average maximum activity at Φ_max_ of 32.3±15.5 (SD) impulses/s during stimulation with dorsally presented polarized light. The distribution of Φ_max_ orientations of the 9 recorded neurons was significantly different from randomness (Rao's spacing test, p<0.01) and ranged from 80° to 140° with an average Φ_max_ of 113°±18° (SD) (Fig. 2J). Stationary polarized light at an *E*-vector orientation of 0° (i.e. near Φ_min_) led to phasic on-inhibition.

A second tangential intrinsic medulla neuron, termed TIM2 was studied in one experiment only. In contrast to TIM1, the branches of TIM2 were concentrated in the DRMe, dorsal regions of the medulla, and in a ventral area of the medulla (Fig. 2E, Movie S2). The cell body of TIM2 was located anteriorly in the optic lobe, dorsomedially from the AMe. Its primary neurite entered the medulla and bifurcated into two main neurites. One neurite projected extensively into dorsal parts of the medulla, and a few branches entered the DRMe. The second main neurite ramified in the ventralmost region of the medulla. No arborizations were observed in the AMe. In contrast to the TIM1 neuron, the polarity of the TIM2 neuron was well defined. Arborizations in the dorsal region of the medulla and in the DRMe were fine (Fig. 2F), suggesting input synapses whereas endings in the ventral region of the medulla were highly varicose (Fig. 2G).

The neuron had a background spike rate of 6.3 impulses/s, and the spike frequency increased to a maximum of 22.6 impulses/s at Φ_max_. Like in TIM1 neurons, spiking activity in TIM2 was sinusoidally modulated during stimulation with a rotating *E*-vector, but did not show polarization opponency, i.e. no *E*-vector orientation inhibited the TIM2 neuron (Fig. 2I). The Φ_max_ angle of the recorded neuron was 4° (Fig. 2J).

### Tangential medulla-lamina neurons

**Figure 3:**
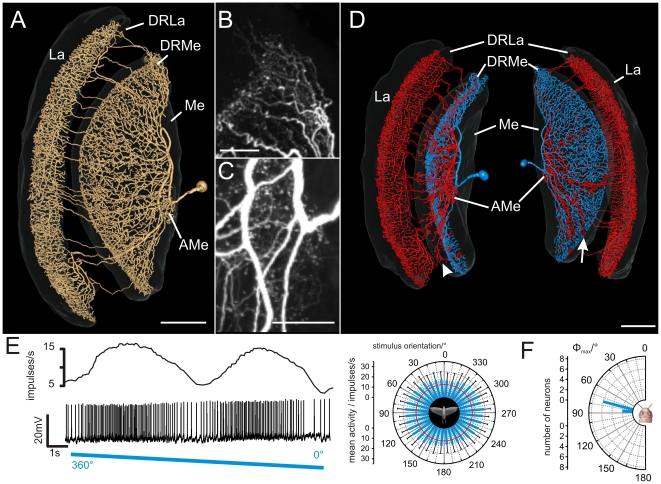
Anatomy and response properties of the tangential medulla-lamina neuron type 1 (TML1). (A) Three-dimensional reconstruction of the TML1 neuron and innervated brain areas (grey, transparent), anterolateral view; AMe, accessory medulla; DRMe, dorsal rim area of the medulla; DRLa, dorsal rim area of the lamina; La, lamina; Me, medulla. (B) Maximum intensity projection of the arborizations of the TML1 neuron in the DRMe. (C) Ramification of the neuron in the AMe, maximum intensity projection of an image stack. (D) Three-dimensional reconstruction of the TML1 neuron shown in A–C; left side: anteromedian view; right side: posteromedian view. Ramifications labeled in blue have smooth fiber terminals. The reconstructed red part of the neuron has a varicose appearance and may, therefore, be the output region of the neuron. Presumed output sites are in medulla layer 1 (white arrowhead) and in the same layer as the presumed inputs (white arrow). (E) Spike train (lower trace) and mean spiking frequency (upper trace) of the TML1 neuron shown in A–D during stimulation with a rotating polarizer (moving average, bin size: 1s). Right panel: Circular diagram of the mean frequency plotted against the stimulus orientation (bin size 10°, n = 12, error bars = SD, Φ_max_ =  84°, Rayleigh test, p = 3.11×10^−9^). The background activity of the neuron is indicated by the red circle. (F) Mean preferred Φ_max_ orientations of the recorded TML1 neurons (n = 8). The values are means from equal numbers of clockwise and counter clockwise rotations of the polarizer. All neurons were recorded in the left medulla. The distribution of Φ_max_ angles differs significantly from randomness (mean Φ_max_ angle: 77°±5.5° (SD); Rao's spacing test, p<0.01; length of mean vector *r* = 0.982). Scale bars: (A,D) 200 µm; (B,C) 50 µm.

Eight recordings were obtained from a tangential medulla neuron with projections to the lamina, termed TML1. The strikingly similar morphology and physiology strongly suggest that all recordings were from the same neuron. TML1 had wide arborizations in the medulla, the DRMe, the AMe, and the lamina (Fig. 3A, Movie S3). The giant soma of TML1 was located anteromedially from the medulla. The primary neurite entered the medulla at the level of the AMe and projected toward dorsal regions of the medulla. Several fibers branched off from the main neurite and gave rise to an extensive meshwork throughout a narrow layer of the medulla and dense ramifications in the DRMe (Fig. 3B). The main neurite made a loop and projected back toward the AMe (Fig. 3C) and the ventral face of the medulla (Fig. 3D, red part of the neuron). Several sidebranches from this looping neurite again entered the narrow medulla layer (Fig 3D, white arrow). Other processes gave rise to beaded terminals in the AMe. Another set of sidebranches from the looping fiber entered the most anterior layer of the medulla with varicose terminals (layer 1, Fig. 3D, white arrowhead). Many of these fibers continued through the first optic chiasm to the lamina. They entered the lamina posteriorly and gave rise to extensive varicose arborizations through the innermost layer of the lamina, but did not invade the dorsal rim area of the lamina (DRLa) (Movie S3). The distinctly different arborizations in different parts of the optic lobe suggest that TML1 neurons receive synaptic input in the medulla and DRMe (Fig. 3D, blue part of the 3D-reconstructed neuron). Possible outputs may be the AMe, layer 1 of the medulla, and the lamina (Fig. 3D, red regions of the neuron).

TML1 neurons had a mean background activity of 7.4±6.2 (SD) impulses/s. Zenithal stimulation with polarized light led to strong tonic excitation, which was modulated sinusoidally during rotation of the polarizer (Fig. 3E). The neurons were maximally activated up to peak frequencies of 26.2±12.4 impulses/s. The preferred *E-*vector orientation of TML1 neurons was significantly different from a uniform distribution (Rao's spacing test, p<0.01). Φ_max_ orientations were tightly clustered between 70° and 90° with a mean of 77°±5.5° (SD) (Fig. 3F).

### Intermedulla neurons

**Figure 4:**
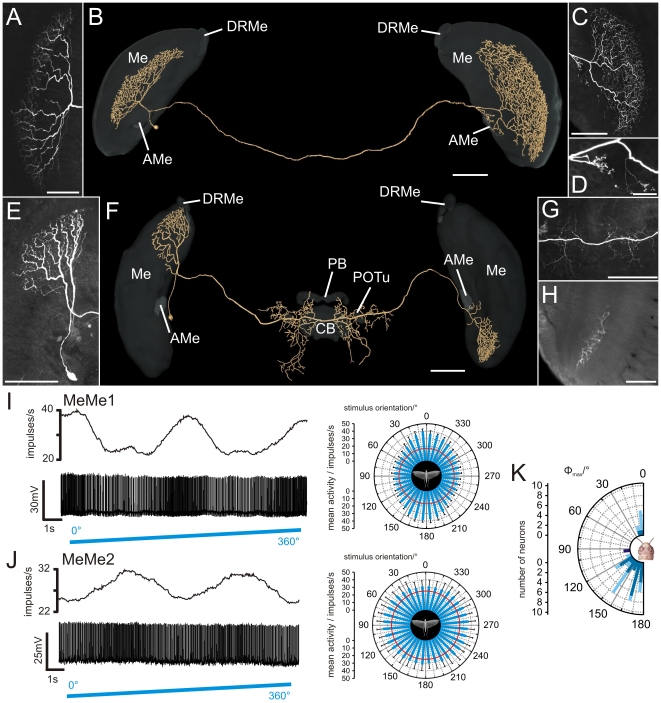
Morphology and physiology of intermedulla neurons (MeMe1, MeMe2). (A) Maximum intensity view of the arborizations of a MeMe1 neuron in the ipsilateral medulla. (B) Three-dimensional visualization of the MeMe1 neuron together with the medulla (Me), accessory medulla (AMe), and dorsal rim area of the medulla (DRMe) (grey, transparent). Anterior view. (C) Ramification of the MeMe1 neuron in the contralateral medulla. (D) A few sidebranches of the MeMe1 neuron arborize in the contralateral AMe. (E) Maximum intensity visualization of a MeMe2 neuron in the ipsilateral medulla. (F) Anterior view of a three-dimensionally reconstructed MeMe2 neuron. Neuropils of the optic lobes and brain areas in the central brain (CB: central body; PB: protocerebral bridge; POTu: posterior optic tubercle) are shown in transparent grey. The neuron did not enter the CB, PB or the POTu. (G) Varicose ramifications of the MeMe2 neuron in the posterior protocerebrum. (H) Axial slice at a depth of about 185 µm shows arborizations of the MeMe2 neuron in the contralateral medulla. (I) Physiology of the MeMe1 neuron, shown in A–D, during stimulation with polarized blue light, rotated in clockwise direction. The neuron showed sinusoidal modulation of spike activity (lower trace) during stimulation with a rotating polarizer. Upper trace: Mean spike frequency (moving average, bin width: 1s). Right panel: mean spike activity of the MeMe1 neuron plotted in a circular diagram (bin size: 10°; n = 10; error bars =  SD; red circle =  background activity; Φ_max_ =  163°; Rayleigh test, p<10^−12^). (J) Response of MeMe2 neuron, shown in E and G to polarized light. Lower lane: spike train of the neuron. Upper lane: mean spike frequency during stimulation (moving average of spike rate in 1s time windows). The right panel shows a circular plot of the mean spike frequency plotted against *E-*vector orientation (bin size 10°, n = 6, error bars =  SD, Φ_max_ =  99°, Rayleigh test, p = 0.037). Red circle represents background activity of the neuron. (K) Distribution of Φ_max_ orientations from 37 MeMe1 neurons and one MeMe2 neuron. Values are plotted for neurons with cell bodies in the left brain hemisphere (n = 24, blue). Values from 13 MeMe1 neurons with cell bodies in the right brain hemisphere were mirrored against the longitudinal axis of the animal (light blue). The orientation of Φ_max_ of all MeMe1 neurons differed significantly from a uniform distribution (mean Φ_max_: 159°±20° (SD); Rao's spacing test, p<0.01; length of mean vector *r*  = 0.784). The recorded MeMe2 neuron had a preferred *E-*vector angle of 99° (dark blue). Scale bars: (A,H) 100 µm; (B,C,E,F,G) 200 µm; (D) 50 µm.

Intermedulla neurons, termed medulla-medulla neurons (MeMe) were recorded in 39 experiments (Fig. 4). MeMe neurons connected the medullae of the right and left hemispheres of the brain. The medulla-medulla neuron 1 (MeMe1) was studied in 37 experiments. All stained neurons had indistinguishable morphology and similar physiological properties again suggesting that all recordings were from the same neuron in different animals. MeMe1 neurons had their soma anteromedially from the medulla in the vicinity of the AMe. Their primary neurite projected posteriorly from the AMe into the ipsilateral medulla (Fig. 4A,B, Movie S4) and bifurcated into two main neurites. One fiber projected into the ipsilateral medulla and gave rise to smooth arborizations in a single layer of the medulla (Fig. 4A). The ramifications did not extend completely throughout the medulla layer but were restricted to an anteromedian region of the layer. The second main neurite left the medulla and ran toward the posterior surface of the optic lobe. It entered the posterior optic tract and commissure to the contralateral optic lobe. There, the fiber turned anteriorly again, entered the medulla and gave rise to a meshwork of varicose processes in a single medulla layer (Fig. 4C). Some varicose sidebranches entered the contralateral AMe (Fig. 4D).

MeMe1 neurons had a mean background spiking rate of 14.1±9.2 (SD) impulses/s in darkness and showed no or only weak polarization opponency (Fig. 4I). Presentation of polarized light at an *E*-vector orientation of 0° from dorsal direction led to phasic on-inhibition that was followed by weak tonic excitation. During rotation of the polarizer the neurons were maximally activated at Φ_max_ up to a mean spike frequency of 33.1±14.3 (SD) impulses/s. The Φ_max_ values of the MeMe1 neurons were significantly different from a random distribution (Rao's spacing test, p<0.01) and had a mean *E*-vector of 159°±20° (SD) (Fig. 4K).

The second type of intermedulla neuron, called medulla-medulla neuron 2 (MeMe2) was encountered in two experiments (Fig. 4E-H, Movie S5). The soma of MeMe2 was located anteriorly close to the AMe. The primary neurite entered the medulla, ran dorsally and ramified in a single layer (Fig. 4E). The arborizations of the neuron were restricted to the dorsalmost region of the medulla and parts of the DRMe. An axonal fiber left the optic lobe posteriorly and projected into the posterior central brain. It gave rise to varicose sidebranches in the median protocerebrum posteriorly from the central complex (Fig. 4F,G). Some processes entered the lateral ocellar tracts. The main neurite continued to the contralateral optic lobe, entered the optic lobe anteriorly, passed the lobula and ran ventrally toward the medulla. It entered the medulla, projected to the ventralmost part of the medulla and arborized in a single medulla layer (Fig. 4H).

The background spiking rate of the MeMe2 neurons ranged from 10.5 to 25.5 impulses/s. Both neurons responded with strong tonic excitation to stimulation with polarized light (*E*-vector at 0°) from the zenith. Sinusoidal modulation of spiking activity during rotation of the polarizer was significant in only one of the two recordings and showed maximum activity of 49.3 impulses/s at Φ_max_ (99°, Fig. 4J,K).

### POL-neurons share innervation of medulla layer 4

To reveal possible sites of synaptic contact between the medulla POL-neurons, we compared the medulla layers that were innervated by the different cell types. Furthermore, we wanted to find out how POL-neurons without synaptic input in the DRMe, like the MeMe1 neurons, receive polarized light information.

**Figure 5:**
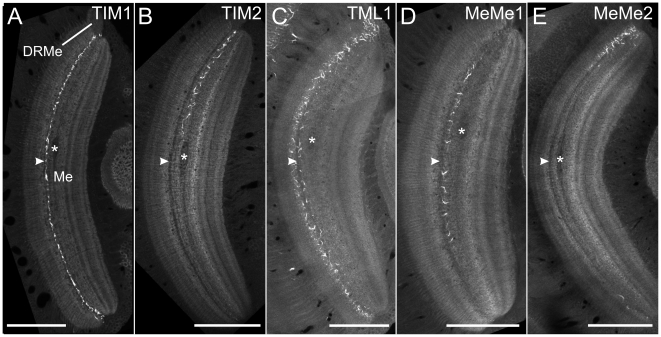
All polarization-sensitive neurons arborize in the same layer of the medulla. (A) Ramifications of the TIM1 neuron (of Fig. 2C) in the medulla. Frontal slice through the medulla at a depth of about 304 µm. (B) Anterior view of the arborizations of the TIM2 neuron at a depth of about 472.5 µm. (C) Axial slice through the medulla and the TML1 neuron at a depth of about 298 µm. (D) Ramifications of the MeMe1 neuron in the ipsilateral medulla at a depth of about 200 µm from the anterior surface. (E) Frontal view of the arborizations of the recorded MeMe2 neuron in the ipsilateral medulla at a level of about 245 µm. Asterisks show a horizontally projecting fiber bundle that traversed the medulla medially from layer 4 and served as an anatomical landmark for the definition of layers. White arrowheads point to a narrow dark sheet that separated layers 3 and 4. All recorded POL-neurons arborized in medulla layer 4 as defined by [33]. Scale bars: 200 µm.

Toward this goal, we rehydrated and sectioned the brain preparations with the dye-injected neurons and labeled the brain sections, in addition, with antibodies against the presynaptic vesicle protein synapsin. This allowed us to identify individual layers of the medulla and to define the medulla layers innervated by the different POL-neurons (Fig. 5). An anatomical landmark of the medulla that simplified the definition of layers was a large dark spot that resulted from a fiber bundle running horizontally along the equator through the medulla (Fig. 5, asterisks). This fiber bundle was located medially from layer 4 of the medulla (as defined by [33]) and facilitated distinction of layers 4 and 5. Another relevant feature was a thin salient dark sheet that separated layers 3 and 4 (arrowheads in Fig. 5).

TIM1 and TIM2 neurons (Fig. 5A, B) and MeMe1 neurons (Fig. 5D) passed the medulla exclusively through layer 4. TML1 and MeMe2 neurons mainly extended via layer 3 but in both cell types numerous sidebranches projected into medulla layer 4 (Fig 5 C,E).

**Figure 6:**
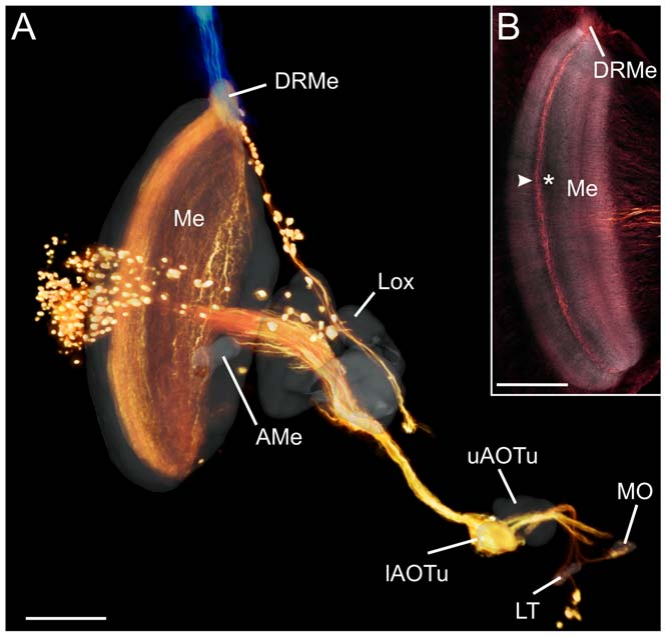
Transmedulla neurons connected to the anterior optic tubercle in the locust brain. (A) Volume rendering visualization of dextran injected into the lower unit of the anterior optic tubercle (lAOTu) revealed the polarization vision pathway from the dorsal rim area of the medulla (DRMe) via the anterior lobula of the lobula complex (Lox) and the AOTu to the lateral triangle (LT) and the median olive (MO) of the lateral accessory lobe. In addition, photoreceptors of the dorsal rim area were stained through injection with dextran conjugated to Alexa488 (blue). Cell bodies of the transmedulla neurons are clustered anteromedially and distally from the medulla. Whether the additional somata and associated fiber bundle dorso-medially from the medulla had ramifications in the DRMe or were stained by leakage of dye into adjacent dorsal areas of the medulla, could not be determined. The innerved brain areas are shown in transparent grey. AMe, accessory medulla; uAOTu, upper unit of the anterior optic tubercle. (B) Axial view of the ramifications of the transmedulla neurons at a depth of 390 µm. Asterisk points to landmark fiber bundle through the medulla; white arrowhead points to dark sheet separating layers 3 and 4. The transmedulla neurons arborize in medulla layer 4, like the recorded POL-neurons. Scale bars: 200 µm.

In addition to the neurons characterized physiologically, we studied the morphology of transmedulla neurons that link the DRMe to the anterior optic tubercle (AOTu) [10]. Owing to the small neurites of these neurons, recordings from transmedulla neurons were not obtained. Injections of biotinylated dextran into the lower unit of the AOTu labeled the polarization vision pathway from the DRMe to the lateral accessory lobe (Fig. 6A). The somata of the transmedulla neurons were clustered near the anterior surface of the optic lobe distally from the medulla. The neurons arborized densely in the DRMe, projected through a distinct layer of the medulla and ran via the anterior optic tract to the anterior lobula and into the AOTu. As in TIM1, TIM2 and MeMe1 neurons, the ramifications of transmedulla neurons within the medulla were restricted to layer 4 (Fig. 6B). Taken together, our data suggest that polarized light information is integrated in the brain via medulla layer 4.

### Azimuth-dependent responses to unpolarized light

In the daylight sky, the ratio between light of long wavelengths (460–700 nm) and light of short wavelengths (300–460 nm) is higher in the solar hemisphere than in the antisolar hemisphere [34]. POL-neurons of the AOTu respond, in addition to polarized light, to unpolarized green and ultraviolet light. Their responses to those stimuli suggest that they could aid in distinguishing the solar and antisolar hemispheres of the sky based on the respective content of wavelengths [16]. Owing to the wide ramifications in the medulla of the POL-neurons studied here, it seemed likely that they, likewise, receive information about the spectral gradient of the sky.

To explore this, we stimulated the POL-neurons of the medulla with an unpolarized green (530 nm) and UV (350 nm) light spot that moved around the locust's head on a circular path at an elevation of 45°. To study whether the tuning to chromatic properties of the sky is hard-wired or requires learning, we performed these experiments on two groups of animals. The first group was raised indoors under artificial illumination, whereas the second group was raised in a greenhouse with open view to the sky.

**Figure 7:**
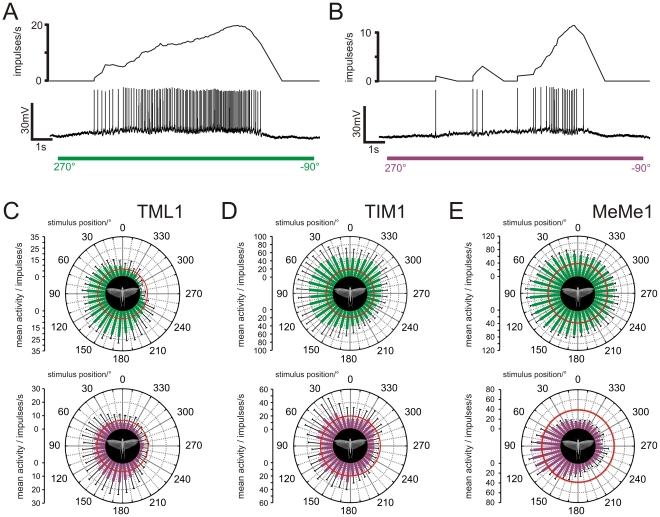
Azimuth-dependent responses of medulla POL-neurons to unpolarized light. All data are from animals raised in a greenhouse. (A,B) Responses of a TML1 neuron to a green and a UV light spot that moved at an elevation of 45° around the head. Upper traces: Mean spike frequency visualized with a moving average bin size of 1s. Lower trace: Spike train. (A) Response of the TML1 neuron to clockwise movement of a green light spot. (B) Response of the same neuron to clockwise movement of a UV light spot. (C–E) Circular diagrams of mean firing rates of medulla POL-neurons plotted against stimulus position. Upper circular plots show responses to a circling green light spot, lower plots, to a circling UV light spot. Bin size of all plots is 10°; error bars =  SD; red circles indicate background activity of the neurons. (C) Responses of a TML1 neuron to a green light spot (n = 6; Φ_max_green = 124°, p<10^−12^) and to a UV light spot (n = 8; Φ_max_UV = 136°, p<10^−12^). (D) Responses of a TIM1 neuron to a green light spot (n = 10; Φ_max_green = 67°, p<10^−12^) and to a UV light spot (n = 8; Φ_max_UV = 91°, p<10^−12^). (E) Responses of a MeMe1 neuron to a green light spot (n = 4) revealed a mean azimuthal direction to green of 97° (p<10^−12^) and during presentation of a UV light spot (n = 4) a mean azimuthal direction to UV of 101° (p<10^−12^).

33 of a total of 36 analyzed neurons (91%) (5 TML1; 4 TIM1; 1 TIM2; 26 MeMe1) responded with a significant azimuth-dependent modulation of spike rate (p>0.05). 12 of these neurons were recorded in animals, reared with direct view of the blue sky (4 TML1; 1 TIM1, 7 MeMe1). During stimulation with a green or UV light spot, all 12 neurons showed an increase in spike rate, when the stimulus was presented ipsilaterally, whereas in the contralateral field of view no response or an inhibition of firing activity was observed (Fig. 7). Two of the four analyzed TML1 neurons responded to both green and UV light in an azimuth dependent way, whereas two other neurons only responded to the green light spot. Stimulation of the TML1 neurons with a circling green light spot led to activation when presented ipsilaterally and to inhibition when presented contralaterally, or it had no effect on the contralateral side (Fig. 7A,C). When stimulating TML1 neurons with UV light, the neurons showed a similar but weaker response (Fig. 7B,C). The TIM1 neuron was tonically activated during the whole stimulus period, but excitation in the ipsilateral visual field was substantially stronger (Fig 7D). The response to the UV light spot was weaker, but again with a higher firing rate when the stimulus was in the ipsilateral visual field. Like the TIM1 neuron, MeMe1 neurons were activated by green light from all azimuthal directions but most strongly in the ipsilateral field of view (Fig. 7E). Stimulation with UV light led to strong inhibition in four MeMe1 neurons when the stimulus was in the contralateral field of view (Fig 7E).

**Figure 8:**
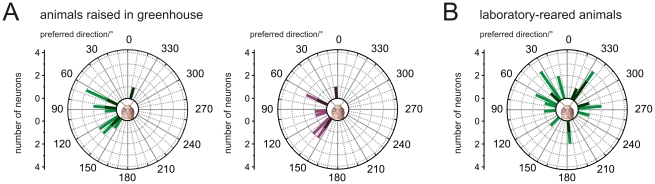
Directional sensitivity to unpolarized light spots in laboratory- and greenhouse-raised animals. (A) Left panel: Distribution of Φ_max_green values of medulla neurons tested in greenhouse-reared animals. Preferred directions are plotted for neurons with cell body in the left brain hemisphere (bright green). Values from neurons with somata in the right brain hemisphere were mirrored (dark green). The distribution of Φ_max_green values differed significantly from randomness (n = 12; Rao's spacing test, p<0.01, length of mean vector *r* = 0.771; mean preferred direction: 97°±41.3° (SD)). Right panel: Distribution of Φ_max_UV values from the same neurons as in the left panel. Values are plotted for cells with somata in the left brain hemisphere (light violet), values from neurons with somata in the right optic lobe were mirrored (dark violet). The distribution differed significantly from randomness (n = 9; Rao's spacing test, p<0.01, length of mean vector *r* = 0.735; mean preferred direction: 103°±45° (SD)). (B) Distribution of Φ_max_green values of medulla POL-neurons analyzed in laboratory-raised animals. Values are plotted for neurons with somata in the left brain hemisphere (light green). Values from neurons with somata in the right brain hemisphere were mirrored against the dorsoventral axis of the animal (dark green). The preferred azimuthal directions of the neurons are distributed randomly (n = 21; Rao's spacing test, p>0.05, length of mean vector *r* = 0.348).

The distribution of the azimuthal tunings to the unpolarized green light spot of all recorded medulla neurons from animals raised in the greenhouse differed significantly from randomness (Fig. 8A, left panel; Rao's spacing test, p<0.01) and had a mean preferred azimuth of 97°±41.3° (SD). The preferred azimuthal tunings to the UV light spot of these neurons also differed significantly from a random distribution (Fig 8A, right panel; Rao's spacing test, p<0.01) with a mean preferred direction at 103°±45° (SD). Thus, the azimuthal tuning of medulla POL-neurons to unpolarized light spots was, unlike that of POL-neurons of the AOTu [16], independent of the wavelength tested. Interestingly, when analyzing the preferred azimuthal direction to unpolarized green light in laboratory-reared animals, the distribution was not significantly different from randomness (Fig 8B). Therefore the azimuthal tuning differed between animals raised with direct view to the blue sky and laboratory-reared animals. In contrast, when comparing the *E*-vector tuning of the medulla neurons between these animals we found no difference between the preferred *E-*vector orientations (Watson-Williams F-test, for MeMe1: F_1,36_ = 0.001; p = 0.972; for TML1: F_1,6_ = 0.442; p = 0.531). Because both groups of animals originated from the same laboratory colony, it is likely that visual experience of the sky had an effect on the azimuthal tuning of medulla POL-neurons in the greenhouse-reared locusts.

### Receptive field structure and ocular dominance

For an appreciation of the relation between polarized light information and azimuthal information, it is important to know the receptive field of the neurons for polarized light. For neurons with a receptive field centered to a point along the solar meridian, the angle between their *E-*vector tuning and their azimuthal tuning to an unpolarized light stimulus-if interpreted as sun - should be 90°. For all other points in the sky the absolute angular difference between those two directions is smaller than 90°. Moreover, owing to diurnal changes of solar elevation, the angular difference between the solar azimuth and the celestial *E*-vector orientation has to be continuously compensated during the course of day [16], [35].

To examine these relations we shifted the *E*-vector stimulus along the left-right meridian to determine the center and bilateral extension of the receptive fields for polarized light in the recorded medulla neurons. In addition, we tested, whether the neurons received monocular or binocular polarized-light input. To define the strength of modulation to polarized light at different positions in the visual field and between ipsi- and contralateral eye stimulation from dorsal direction, we calculated the response amplitude value R. For a comparison of different recordings, R values were normalized to the largest response value or, in case of ocular dominance, to the response to dorsal stimulation of both eyes.

**Figure 9:**
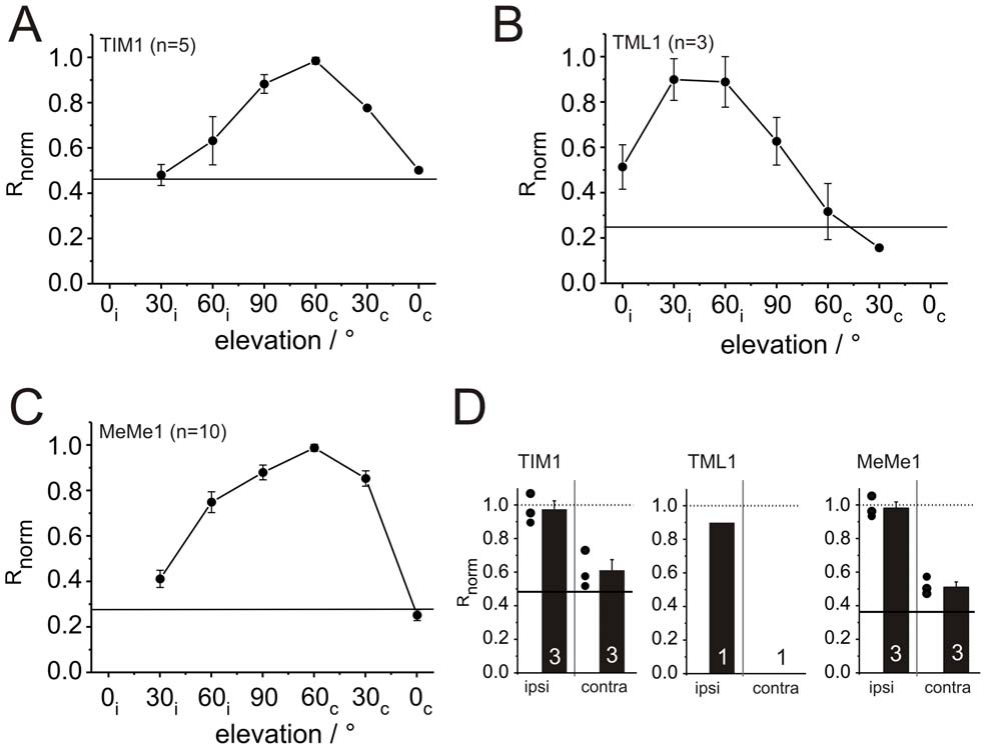
Receptive field size and position along the left-right meridian and ocular dominance of medulla POL-neurons. (A–C) Receptive fields of the neurons. Response amplitudes R were measured at particular elevations along the left-right meridian and were normalized to the largest R value (R_norm_). For better visualization, the averaged R_norm_ values were connected by lines. Error bars indicate standard error. The terms ipsilateral (i) and contralateral (c) were defined with respect to the position of the soma of the analyzed cell. Horizontal lines indicate normalized background variability of the neurons, measured in a section of the spike train without stimulation. (A) Averaged receptive field of TIM1 neurons (n = 5). The receptive field is centered to an elevation of 60° contralaterally. (B) Receptive field of TML1 neurons (n = 3). The center of the receptive field is positioned in the ipsilateral field of view. (C) The receptive field center of MeMe1 neurons (n = 10) is located at an elevation of 60° contralaterally. (D) Ocular dominance of the medulla neurons. The R values were calculated for zenithal monocular stimulation of the ipsilateral (ipsi) and contralateral eye (contra) and were normalized to the R value during stimulation of both eyes. The averaged R_norm_ values for monocular stimulation are shown as bars (error bars = SE). In addition, in the TIM1- and MeMe1-diagram the normalized R values of the individual neurons are shown as black dots. Stimulation of both eyes (R_norm_  = 1.0) is indicated as dashed lines. Mean background variability of the analyzed TIM1- and MeMe1-neurons is shown as solid black line.

The receptive field for polarized light of TIM1 neurons was investigated in five animals. It was centered at an elevation of 60° contralaterally and had a width of about 85° along the left-right meridian (Fig. 9A). In contrast to TIM1 neurons, the receptive field of TML1 neurons was centered to the ipsilateral side at an elevation between 30° and 60° (Fig. 9B). The width of the receptive field of the TML1 neurons was about 90°. The receptive field along the left-right meridian of MeMe1 neurons was studied in ten experiments. They had a large receptive field of about 110° along the left-right meridian which was, like that of TIM1 neurons, centered at an elevation of 60° contralaterally (Fig. 9C). In all three cell types, *E*-vector tuning (Φ_max_) did not differ systematically at different positions along the left-right meridian (not shown).

The ocular dominance of three TIM1, one TML1 and three MeMe1 neurons was analyzed by monocular stimulation of the ipsi- and contralateral eye (Fig. 9D). TIM1 and MeMe1 neurons showed a substantial difference in the response strength between stimulation of the ipsilateral and contralateral eye (Fig 9D). In addition, the response to stimulation of both eyes did not differ from the response to stimulation of only the ipsilateral eye. Ocular dominance of the TML1 neuron was investigated in one animal. Whereas stimulation of the ipsilateral eye resulted in a strong sinusoidal modulation, no spiking activity occurred during stimulation of the contralateral eye with zenithal polarized light.

### No evidence for solar elevation compensation in the medulla

**Figure 10:**
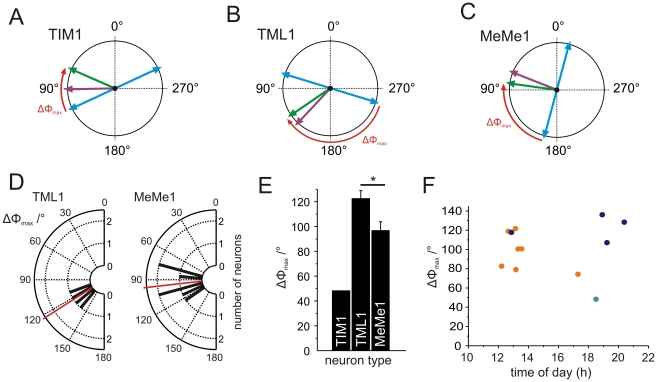
Relationship between tuning to zenithal polarized blue light and azimuthal tuning to unpolarized light in medulla POL-neurons of greenhouse-raised animals. (A–C) Tuning of individual neurons to polarized light (blue bidirectional arrows) and to unpolarized light (green light: green arrows; UV light: violet arrows). Red arrows indicate the calculated angles between the preferred *E*-vector orientation and preferred azimuthal direction (ΔΦ_max_) shown in D–F. (A) TIM1 neuron. (B) TML1 neuron. (C) MeMe1 neuron. (D) Distribution of the angular differences between *E-*vector tuning and azimuthal green-light tuning (ΔΦ_max_) of the TML1 (n = 4) and the MeMe1 neurons (n = 7). Red lines show the averaged ΔΦ_max_ values. In both neurons the distribution of ΔΦ_max_ values differs significantly from randomness (bin size: 10°; Rao's spacing test, p<0.01). (E) The averaged ΔΦ_max_ values of the TIM1 neuron, the TML1 neurons (n = 4) and the MeMe1 neurons (n = 7). ΔΦ_max_ differs significantly between TML1 neurons and MeMe1 neurons (Watson-Williams F-test, F_1,9_ = 6.095; p = 0.036). (F) The ΔΦ_max_ values of the medulla neurons plotted against time of day of the recording. Orange dots indicate ΔΦ_max_ values of MeMe1 neurons, blue dots the ΔΦ_max_ values of TML1 neurons, and the green dot, the ΔΦ_max_ value of the TIM1 neuron.

Because the receptive fields of the medulla neurons for polarized light are not centered to the zenith, the angle between the solar azimuth and the neurons' preferred *E*-vector orientation (ΔΦ_max_) becomes increasingly different from 90° with increasing solar elevation. Assuming that the green light spot is interpreted as the sun, we therefore calculated the angular relation and its daytime dependence between *E-*vector tuning to polarized blue light and azimuthal tuning to green light of neurons recorded from animals raised in the greenhouse (ΔΦ_max_, Fig. 10A–C). In these animals, ΔΦ_max_ values ranged from about 48° in the TIM1 neuron to about 140° in TML1 neurons. The distribution of ΔΦ_max_ values in MeMe1 neurons and TML1 neurons was significantly different from randomness (Fig. 10D, Rao's spacing test, p<0.01). The ΔΦ_max_ values of MeMe1 neurons were clustered between 70° and 120° whereas the ΔΦ_max_ distribution of TML1 neurons ranged from 100° to 140°. Furthermore, the ΔΦ_max_ values differed significantly between the TML1 neurons and the MeMe1 neurons (Fig. 10E, Watson-Williams F-test, F_1,9_ = 6.095; p = 0.036). Whereas the TML1 neurons had a mean ΔΦ_max_ value of 122.6°±12.5° (SD) (Fig. 10B,E), the mean ΔΦ_max_ in MeMe1 neurons was 96.6°± 18.7° (SD) (Fig. 10C,E). To examine, whether ΔΦ_max_ changed during the day, we plotted the ΔΦ_max_ values against the time of day when the recordings were performed (Fig. 10F). If the neurons compensated the changing *E*-vector-orientation during the day in a way similar to neurons of the AOTu, ΔΦ_max_ values should be low at noon and rise towards the evening [16]. Surprisingly, the medulla neurons did not show daytime-dependent changes in their ΔΦ_max_ (Fig. 10F). The values were similar at noon and in the evening although the neurons were recorded during the same time of year as those of the AOTu. Furthermore, the response strength (R values) of the neurons did not differ significantly over the course of the day (not shown). The experiments suggest that circadian signals that are essential to compensate celestial compass cues for daytime changes in solar elevation are integrated into the polarization vision system at a processing stage between TML1/MeMe1 neurons and neurons of the AOTu.

**Figure 11:**
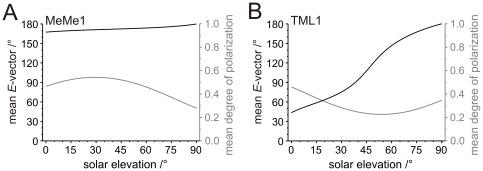
Models of mean *E-*vector and mean degree of polarization within the receptive field of MeMe1- and TML1 neurons at different elevations of the sun. The models were calculated according to the single scattering Rayleigh model [25]. The models are based on circular receptive fields with a diameter of 110° for MeMe1 neurons and 90° for TML1 neurons, corresponding to their width along the left-right meridian. Receptive field centers were defined according to response amplitudes along the left-right meridian shown in Fig. 9 (MeMe1: 60° contralateral; TML1: 45° ipsilateral). The Φ_max_green values (MeMe1: 77.5°±39.6° (SD); TML1: 133.27°±7.6° (SD)) were taken as the respective solar azimuths. (A) Mean *E-*vector orientation (black line) and mean polarization degree (grey line) in the receptive field of MeMe1. (B) Mean *E-*vector orientation (black line) and mean degree of polarization (grey line) in the receptive field of TML1 plotted against solar elevation.

To estimate changes in spiking activity with changing solar elevation, we calculated the mean *E*-vector and the mean degree of polarization within the receptive field of MeMe1 and TML1 neurons at different solar elevations (Fig. 11). Both calculations were performed for a solar azimuth identical to the Φ_max_green value (MeMe1: 77.5°±39.6° (SD); TML1: 133.27°±7.6° (SD)). The receptive field centers for polarized light were assumed as 60° contralateral for MeMe1 and 45° ipsilateral for TML1 (Fig. 9B,C).

In MeMe1 neurons, the mean *E*-vector within the receptive field was relatively constant (between 165° and 180°) at all solar elevations (Fig. 11A) and close to the mean Φ_max_ for polarized light of MeMe1 (159°). The mean degree of polarization ranged from 0.28 to 0.54 with a maximum at 30°. Therefore, MeMe1 should respond most strongly when the sun is ipsilaterally at an elevation of 30°. This coincides with the strongest *E*-vector at an elevation of 60° contralaterally. In TML1 neurons the elevation of the sun had a substantial effect on the mean *E-*vector orientation, owing to the ipsilateral position of the receptive field for polarized light (Fig. 11B). At low solar elevations (0°–30°) the degree of polarization was highest, and the mean *E*-vector within the receptive field was close to Φ_max_ of TML1 (77°). With higher solar elevations, the degree of polarization became lower and the mean *E*-vector differed increasingly from the *E*-vector tuning of TML1 (Fig. 11B). This should result in an increasingly smaller contribution of the polarization channel to spiking activity in TML1 at higher solar elevations.

## Discussion

Intracellular recordings revealed five types of POL-neuron with ramifications in the medulla of the desert locust. Some of these neurons had additional processes in the AMe and/or the DRMe. In addition to responses to polarized light, the neurons showed azimuth-dependent responses to green and UV light spots. Outdoors, the most likely sources of these stimuli will be the sky polarization pattern and the intensity gradient and chromatic contrast in the sky. Ramifications of all neurons in medulla layer 4 suggest that this layer integrates signals from the polarization pattern of the sky with unpolarized celestial cues for sky compass signaling.

### POL-neurons in the locust medulla

Four neurons, TIM1, TML1 and MeMe1 and MeMe2, were studied through multiple recordings. In the TIM1, TML1, and MeMe1 neurons the distributions of Φ_max_ were different from randomness. The preferred *E*-vector orientations of the neurons were clustered narrowly around their average Φ_max_. Combined with the striking similarities in morphologies, this suggests that TIM1, TML1 and MeMe1 and MeMe2 neurons occur as single neurons per brain hemisphere. TML1- and MeMe1 neurons were reported previously by [36] as “medulla tangential with lamina projections” (TML1) and “medulla tangential with contralateral optic-lobe projections” (MeMe1). Furthermore, the TIM1 cell was described by [29] as “intrinsic medulla neuron”. Those studies, however, presented data from single recordings only with highly incomplete characterization. In another species, the cricket *Gryllus campestris*, medulla POL-neurons were studied extensively [37]–[39]. One type, termed POL1, resembles MeMe1 cells but has additional ramifications in the ipsilateral AMe and DRMe [38]. In contrast to MeMe1, POL1-neurons showed strong polarization opponency and occurred as three subtypes with Φ_max_ around 0°, 60° and 120° [38], [40]. A second type of POL-neuron in the cricket brain, termed POL3, had arborizations restricted to the medulla and AMe [24] similar to the locust TIM1 neuron but, unlike TIM1, POL3 again showed polarization opponency. It therefore appears that a similar inventory of POL-neurons is present in crickets and locusts, however with species-specific differences in morphologies and physiological properties.

Although not studied physiologically, transmedulla neurons are likely to transmit polarized-light information from the medulla to the AOTu [10]. Arborizations of the TIM1, TIM2 and MeMe1 neurons were, like those of the transmedulla neurons, confined to layer 4 of the medulla. TML1 and MeMe2 neurons mainly arborized in layer 3 but entered layer 4 with numerous sidebranches. Layer 4 of the medulla may, therefore, be specialized to integrate sky compass information in the brain.

### Sensitivity to polarized light

Behavioral studies in tethered flying locusts showed that polarotaxis is mediated by photoreceptors in the DRA [4]. The DRA consists of ommatidia with optical axes directed upwards and up to 30° to the contralateral side [6]. To avoid interference with the color vision system, DRA photoreceptors are homochromatic with highest sensitivity in the blue range [41]. In each DRA ommatidium, photoreceptor microvilli are arranged in two blocks of orthogonal orientation [6]. This has been suggested to be the basis for polarization opponency found in POL-neurons of crickets [42] and in central-brain neurons of locusts [8]. In contrast, most medulla neurons studied here were excited at Φ_max_ but lacked an orthogonal inhibitory input. This could mean that these neurons, perhaps unlike transmedulla neurons, are not a direct link in the polarization vision pathway, but rather modulate polarization vision in the medulla.

TIM1, TML1 and MeMe1 neurons received polarized light information from the ipsilateral eye. Therefore, the commissural MeMe neurons do not provide input to the contralateral TIM1, TML1 and MeMe1 neurons. Corresponding to the contralaterally directed optical axes of DRA photoreceptors [6], the receptive fields of the TIM1 and MeMe1 neurons were centered at an elevation of 60° contralaterally. Unexpectedly, the TML1 neuron had a receptive field centered in the ipsilateral hemisphere. Similar to some central-complex neurons and descending neurons [12], [15], TML1 was still strongly polarization sensitive when the stimulus was at the ipsilateral equator (0° elevation), a position that can hardly be detected by DRA photoreceptors. It is, therefore, likely that those responses were mediated by photoreceptors in the lateral eye. Behavioral experiments reported that locusts detect polarized reflections of water surfaces with ventral eye regions [43], which is known particularly for insects living near water surfaces such as backswimmers [44]. Taken together, the data suggest the existence of eye regions in locusts that extend the field of view for polarized light toward ventral directions.

### Sensitivity to chromatic stimuli

All neurons had extensive arborizations in the medulla, and these sites probably mediated their responses to the unpolarized lights. Neurons of the AOTu, likewise, responded to unpolarized light and showed color-opponent responses to UV and green light [45] which was argued to aid in the discrimination between the solar and antisolar hemispheres [16]. POL-neurons of the medulla did not show color opponency. Instead their azimuthal tuning to UV and green light was strikingly similar in the ipsilateral field of view. Wavelength independence was also observed in the spectral responses of POL-neurons of the monarch butterfly [35], suggesting that monarch butterflies use the solar azimuth itself as a celestial cue. Color opponency in AOTu neurons, therefore, most likely results from convergence of specific color-coding and polarization-coding transmedulla neurons in the AOTu.

Green light induced strong tonic excitation when stimulating the ipsilateral eye. In contrast, ultraviolet light elicited inhibition at the contralateral eye. Because polarization input in medulla neurons is mediated through the ipsilateral eye, contralateral UV inhibition must be provided through commissural neurons that are not sensitive to polarized light. Based on these data it will be interesting to analyze spatial tuning to spectral cues in the central complex. Central-complex neurons receive bilateral polarization input and have zenith-centered receptive fields for polarized light [12]. We, therefore, predict that these neurons receive a combination of spatially opponent and color opponent chromatic input for a solar elevation-independent compass signal.

Rearing conditions had a strong effect on the azimuthal chromatic tuning of the medulla POL-neurons. Likewise, rearing of locusts with or without direct view to the blue sky had considerable effect on the *E-*vector tuning of AOTu neurons [16]. Both observations suggest that meaningful integration of sky chromatic and polarization signals strongly depends on visual experience of the sky during development.

### Integration of sky compass cues and circadian clock

The medulla POL-neurons showed cell-type specific tuning to dorsal polarized light and to a particular azimuth of unpolarized light. In contrast to POL-neurons of the AOTu, the next central stage in the polarization vision pathway, we found no evidence for daytime dependent changes in tuning characteristics in medulla TML1 and MeMe1 neurons. Therefore, the signaling strength in medulla POL-neurons does not only depend on solar azimuth, but also on solar elevation. Modeling sky polarization in the receptive fields of MeMe1 and TML1 at solar azimuths corresponding to Φ_max_green shows that the *E*-vector response of TML1 will change much more dramatically with changing solar elevation than that of MeMe1 (Fig. 11). In contrast, the spike rate of TML1 should be less sensitive to changes in solar azimuth, because the high degree of polarization in the ipsilateral sky will provide substantial input when the sun is in the contralateral (non-preferred) hemisphere. An important function of the medulla neurons studied here probably lies in the communication between the sky compass and the circadian systems. Both TML1 and MeMe1 neurons had varicose and, therefore, presumably output processes in the AMe, the presumptive circadian clock of the brain [30], [46]. The different dependencies of medulla neurons on solar elevation and solar azimuth might, therefore, provide a highly differentiated zeitgeber signal to the circadian clock and could also contribute to photoperiodic timing, which is probably associated with the circadian clock [47]. If communication of the medulla with the AMe is bidirectional, as suggested by the ramifications of TIM1 in the AMe, the circadian clock could, on the other hand, directly influence the integration of sky polarization and chromatic information at the entrance to the AOTu.

## Supporting Information

Movie S1 **Movie presenting a 360° vertical rotation of the three-dimensional reconstruction of the TIM1 neuron shown in** **Figure 2D** **.** The innerved brain areas are shown in transparent grey.
(AVI)

Movie S2 **Movie of the 3D reconstructed TIM2 neuron illustrated in** **Figure 2E** **.** The medulla is shown in transparent grey.
(AVI)

Movie S3 **Short movie of the ramification of the TML1 neuron in the medulla (transparent grey) reconstructed in three-dimensions (same neuron as in** **Figure 3A and D** **).**
(AVI)

Movie S4 **Movie illustrating the three-dimensionally reconstructed MeMe1 neuron of** **Figure 4B** **and the medullae of both hemispheres (transparent grey) rotating around the vertical axis.**
(AVI)

Movie S5 **Movie showing a 3D model of the MeMe2 neuron (same as in** **Figure 4F** **).** Optic lobe neuropils and brain areas of the central brain are shown in transparent grey. Vertical rotation.
(AVI)
